# The Use of Gabapentin to Decrease Morphine Consumption After Surgical Debridement for Burns

**DOI:** 10.5812/atr.6468

**Published:** 2012-08-21

**Authors:** Gerard Ee, Rachel Ho

**Affiliations:** 1 National University Hospital, Singapore; 2 Singapore General Hospital, Singapore

**Keywords:** Gabapentin, Burns, Morphine

## Dear Editor,

I read with great interest the recent manuscript by Siamak Rimaz on “ Effect of Gabapentin on Morphine Consumption and Pain after Surgical Debridement of Burn Wounds: A Double-Blind Randomized Clinical Trial Study” published in Archives of Trauma Research (1). In this article they recruited 50 patients and through a randomized, double-blind, placebo-controlled study demonstrated a significant improvement in pain scores and clinical parameters after administration of a single oral dose of 1200mg gabapentin.

The family of opioid analgesics has been the backbone of analgesia in burn patients. However, it is well known that this group of drugs tend to have numerous side effects and recent data has implicated them as being capable of inducing pain N-methyl-D-aspartate (NMDA) receptor antagonists such as ketamine and gabapentin have recently became popular due to the marked opiate sparing effects. The effect of pain modulation on the descending pathways with the use of gabapentin has been shown in numerous studies (2, 3). Indeed I believe that the pain post-mastectomy is different from a post surgical debridement of burn wounds, accounting for the difference in clinical results in studies comparing the effect of gabapentin in different procedures. The authors have recorded down respiratory rate as one of the clinical indicators of pain. Despite no significant difference was demonstrated between the gabapentin group and the placebo group, I note that there was a decrease in respiratory rate at all-time points in the gabapentin group. I understand the rational of using these parameters as a mean in recording pain but would one not expect the increased use of opiates in the placebo group to result in a greater influence on respiratory depression? It would be worth commenting on this. Lastly, the authors have focused on the analgesic effect of gabapentin in burn patients. However, it would worth to mention the effect of gabapentin on post-burn pruritus as well. Post-burn pruritus has a reported incidence of between 80-100% and gabapentin has been proposed to have a modulating effect on this distressing symptom. It would be interesting to study if the Gabapentin group demonstrated better relief than the standard antipruritic protocols. 
